# A note on the almost-Schur lemma on smooth metric measure spaces

**DOI:** 10.1186/s13660-018-1791-y

**Published:** 2018-07-27

**Authors:** Jui-Tang Chen

**Affiliations:** Department of Mathematics, National Taiwan Normal University, Taipei City, Taiwan, R.O.C.

**Keywords:** 58J50, 53C23, 53C21, 53C24, Almost-Schur inequality, Einstein manifold, Smooth metric measure space

## Abstract

In this paper, we prove almost-Schur inequalities on closed smooth metric measure spaces, which implies the results of Cheng and De Lellis–Topping whenever the weighted function *f* is constant.

## Introduction

In 2012, De Lellis and Topping [11] proved an almost-Schur lemma; that is, if a closed Riemannian manifold has nonnegative Ricci curvature, an almost-Schur inequality involves scalar curvature and Ricci curvature:

1.1 $$ \int_{M} ( R-\overline{R} ) ^{2}\,dv\leq \frac{4n ( n-1 ) }{ ( n-2 ) ^{2}} \int_{M} \biggl\vert \mathit{Ric}-\frac{R}{n}g \biggr\vert ^{2}\,dv. $$

In particular, the equality holds if and only if this manifold is Einstein and has constant scalar curvature.

In [9], Ge and Wang proved the almost-Schur lemma under the condition of nonnegative scalar curvature in a four-dimensional Riemannian manifold.

In [6], Cheng considered closed Riemannian manifolds with negative Ricci curvature and obtained a generalization of the De Lellis–Topping type inequality. That is, if $\mathit{Ric}\geq- ( n-1 ) K$ for some constant $K\geq0$, she showed that

1.2 $$ \int_{M} ( R-\overline{R} ) ^{2}\,dv\leq \frac{4n ( n-1 ) }{ ( n-2 ) ^{2}} \biggl( 1-\frac{nK}{\lambda_{1}} \biggr) \int _{M} \biggl\vert \mathit{Ric}-\frac{R}{n}g \biggr\vert ^{2}\,dv, $$

where $\lambda_{1}$ is the first non-zero eigenvalue of Laplacian on $( M,g ) $. For more references, see [3–5, 7, 10, 18].

In this paper, we study De Lellis–Topping type inequality on a smooth metric measure space. First, we recall some definitions of smooth metric measure space.

For an *n*-dimensional closed Riemannian manifold $( M^{n},g ) $ and a smooth function *f* on *M*, a triple $( M^{n},g,dv_{f} ) $ is a smooth metric measure space with a weighted volume identity $dv_{f}=e^{-f ( x ) }\,dv$, where *dv* is the volume element of *M* with respect to the metric *g*. Let $( \nabla f\otimes\nabla u ) _{ij}=\frac{1}{2} ( f_{,i}u_{,j}+f_{,j}u_{,i} ) $, and let *Hess* be the Hessian under the metric *g*. We define the weighted Laplacian by the trace of

$$( \mathit{Hess}_{f}\,u ) _{ij}\equiv ( \mathit{Hess}\,u ) _{ij}- ( \nabla f\otimes\nabla u ) _{ij}; $$

that is,

$$\Delta_{f}u=\Delta u- \langle\nabla f,\nabla u \rangle, $$

and it is a self-adjoint operator concerning $dv_{f}$.

Consider the *m*-Bakry–Émery and ∞-Bakry–Émery Ricci tensor on a smooth metric measure space by

$$\mathit{Ric}_{f}^{m}=\mathit{Ric}+\mathit{Hess}\,f- \frac{1}{m}\nabla f\otimes\nabla f,\quad m>0, $$

and

$$\mathit{Ric}_{f}=\mathit{Ric}+\mathit{Hess}\,f, $$

respectively. If $\mathit{Ric}_{f}=\lambda g$ (or $\mathit{Ric}_{f}^{m}=\lambda g$) for some $\lambda\in\mathbb{R}$, then *M* is quasi-Einstein (or *m*-quasi-Einstein). In particular, if *f* is a constant function, then *M* is Einstein.

According to the classical Bochner’s formula, we have a similar formula

$$\frac{1}{2}\Delta_{f} \vert \nabla u \vert ^{2}= \vert \mathit{Hess}\,u \vert ^{2}+ \langle\nabla u,\nabla \Delta_{f}u \rangle +\mathit{Ric}_{f} ( \nabla u,\nabla u ) $$

for $u\in C^{3} ( M ) $ on *M*. Therefore, many results have been extended from Riemannian manifolds to smooth metric measure spaces. We refer the reader to [1, 2, 8, 13–18] for further references.

The paper is organized as follows. In Sect. 2, we show our main results. In particular, the proofs of Theorems 2.1 and 2.2 are shown in Sect. 2.1. In Sect. 2.2, we prove Theorem 2.3 and show partial results for the open problem. Finally, we provide a conclusion in Sect. 3.

## Results and discussion

First, we show the work by Wu [18], which is a type of inequality for an almost-Schur lemma on smooth metric measure spaces. Let

$$N_{f}^{m}\equiv \biggl( R+\frac{2 ( m-1 ) }{m}\Delta f- \frac{m-1}{m} \vert \nabla f \vert ^{2} \biggr) e^{-\frac{2}{m}}$$

and

$$\overline{N}_{f}^{m}=\frac{\int_{M}N_{f}^{m}\,dv_{f}}{\int_{M}\,dv_{f}}$$

for any positive number $m>2$; if

$$\mathit{Ric}_{f}^{m}\geq\frac{ \vert \nabla f \vert ^{2}}{m}g, $$

then

$$\int_{M} \bigl( N_{f}^{m}- \overline{N}_{f}^{m} \bigr) ^{2}e^{-f}\,dv \leq \frac{4 ( m+1 ) ( m-2 ) }{m^{3}} \int_{M} \biggl\vert \mathit{Ric}_{f}^{m}+ \frac{\operatorname{tr}\mathit{Ric}_{f}^{m}}{m-2}g \biggr\vert ^{2}e^{-\frac{m+4}{m}f}\,dv. $$

Moreover, the equality holds if and only if

$$\mathit{Ric}_{f}^{m}+\frac{\operatorname{tr}\mathit{Ric}_{f}^{m}}{m-2}g=0. $$

Thus, he generalized De Lellis and Topping’s result.

From Wu’s work, we want to improve the inequality that is an expansion of the almost-Schur inequality (1.1) for more general Ricci curvature conditions.

In this paper, for convenience, unless otherwise specified, we provide some notation as follows:

$$\textstyle\begin{cases} R_{f}=R+\Delta f, \qquad V_{f}(M)=\int _{M}\,dv_{f}, \\ \overline{R}=\frac{\int_{M}R\, dv_{f}}{V_{f}(M)}, \qquad \overline{R_{f}}=\frac{\int_{M}R_{f}\,dv_{f}}{V_{f}(M)}, \\ R\mathring{i}c=\mathit{Ric}-\frac{R}{n}g, \qquad R\mathring {i}c_{f}=\mathit{Ric}_{f}-\frac{R_{f}}{n}g. \end{cases} $$

Now we state our results.

### Theorem 2.1

*Let* $( M^{n},g,dv_{f} ) $, $n>2$, *be a closed smooth metric measure space*. *If*

$$\mathit{Ric}_{f}\geq \bigl( \Delta f- ( n-1 ) K \bigr) g, $$

*then*

2.1 $$ \Vert R_{f}-\overline{R_{f}} \Vert _{L^{2}}\leq \frac {2n\sqrt{A}}{n-2} \Vert R\mathring{i}c_{f}-\mathit{Hess}\,f \Vert _{L^{2}}+ \Vert \Delta f \Vert _{L^{2}}, $$

*where* $\Vert \cdot \Vert _{L^{2}}^{2}=\int_{M}|\cdot|^{2}\,dv_{f}$,

$$A=\frac{n-1}{n}+\frac{ ( n-1 ) K}{\lambda_{1}}, $$

*and* $\lambda_{1}$ *is the first positive eigenvalue of the weighted Laplacian* $\Delta_{f}$. *Moreover*, *the equality holds if and only if* *M* *is Einstein and has constant scalar curvature with respect to the metric* *g*.

### Theorem 2.2

*Let* $( M^{n},g,dv_{f} ) $, $n>2$, *be a closed smooth metric measure space*. *If*

$$\mathit{Ric}_{f}^{m}\geq \biggl( \frac{1}{m} \vert \nabla f \vert ^{2}- ( n-1 ) K \biggr) g $$

*for any positive constant* *m*, *then*

2.2 $$ \int_{M} ( R-\overline{R} ) ^{2}\,dv_{f} \leq\frac {4n^{2}A}{ ( n-2 ) ^{2}} \int_{M} \vert R\mathring{i}c \vert ^{2} \,dv_{f}, $$

*where*

$$A=\frac{n-1}{n}+\frac{m}{2}+\frac{ ( m+2 ) ( n-1 ) K}{2\lambda_{1}}, $$

*and* $\lambda_{1}$ *is the first positive eigenvalue of the weighted Laplacian* $\Delta_{f}$. *Moreover*, *the equality holds if and only if* *M* *is Einstein and has constant scalar curvature with respect to the metric* *g*.

### Theorem 2.3

*Let* $( M^{n},g,dv_{f} ) $, $n>2$, *be a closed smooth metric measure space*. *If*

$$\mathit{Ric}_{f}\geq \bigl( \Delta f- ( n-1 ) K \bigr) g, $$

*then*

2.3 $$ \int_{M} ( R-\overline{R} ) ^{2}\,dv_{f} \leq\frac {4n^{2}A}{ ( n-2 ) ^{2}} \int_{M} \vert R\mathring{i}c \vert ^{2} \,dv_{f}, $$

*where*

$$A=\frac{n-1}{n}+\frac{ ( n-1 ) K}{\lambda_{1}}, $$

*and* $\lambda_{1}$ *is the first positive eigenvalue of the weighted Laplacian* $\Delta_{f}$.

### Remark 2.1

Inequality (2.1) in Theorem 2.1 is sharp in the sense of two aspects. One is that the constant

$$\frac{2n\sqrt{A}}{n-2}=\sqrt{\frac{4n^{2}A}{ ( n-2 ) ^{2}}}=\sqrt{\frac{4n ( n-1 ) }{ ( n-2 ) ^{2}} \biggl( 1-\frac{nK}{\lambda_{1}} \biggr) }$$

is equal to the square root of the constant in inequality (1.2), then this inequality implies inequality (1.2) whenever *f* tends to a constant. The other is that if the equality of (2.1) holds, then *M* is Einstein and has constant scalar curvature with respect to the metric *g*.

### Remark 2.2

In Theorem 2.3, inequality (2.3) is almost the same as inequality (1.2). If the equality of (2.3) holds, “*M* is trivial Einstein and has constant scalar curvature” remains an open problem. We also note that, due to the work of Cheng [6], we have a partial result about this topic (see Sect. 2.2).

### Proofs of Theorems 2.1 and 2.2

First, it is easy to verify that in Theorems 2.1, 2.2, 2.3 we may select *f* such that $\int f\,dv_{f}=0$ since (2.1), (2.2), and (2.3) are valid whenever we replace *f* with $f-\bar{f}$, where $\bar {f}=\frac{\int_{M}f\,dv_{f}}{V_{f}(M)}$.

#### Proof of Theorem 2.1

Assume that *R* is the nontrivial scalar curvature on *M* with respect to metric *g*, and $R_{f}=R+\Delta f$. According to the Sobolev embedding theorem and calculus variation, there exists a nontrivial solution $u:M\rightarrow R$ of the equation

2.4 $$ \textstyle\begin{cases} \Delta_{f}u=R_{f}-\overline{R_{f}}, \\ \int_{M}u\,dv_{f}=0, \end{cases} $$

where

$$\overline{R_{f}}=\frac{\int_{M}R_{f}\,dv_{f}}{V_{f}(M)}. $$

We also note that the second Bianchi identity $\operatorname {div}\mathit{Ric}=\frac {1}{2}\nabla R$ implies

$$\begin{aligned} ( \operatorname{div}\mathit{Ric}_{f} ) _{j} = & ( \operatorname{div}\mathit{Ric} ) _{j}+ ( \operatorname {div} \mathit{Hess}\,f ) _{j} \\ = & \nabla_{i}R_{ij}+ ( \operatorname{div}\mathit{Hess} \,f ) _{j} \\ = & \frac{1}{2}R_{j}+ ( \operatorname{div}\mathit{Hess}\,f ) _{j} \\ = & \frac{1}{2}R_{f,j}-\frac{1}{2} ( \Delta f ) _{j}+ ( \operatorname{div}\mathit{Hess}\,f ) _{j}; \end{aligned}$$

therefore,

$$\begin{aligned} ( \operatorname{div}R\mathring{i}c_{f} ) _{j} = & ( \operatorname{div}\mathit{Ric}_{f} ) _{j}-\frac{R_{f,j}}{n} \\ = & \frac{n-2}{2n}R_{f,j}-\frac{1}{2} ( \Delta f ) _{j}+ ( \operatorname{div}\mathit{Hess}\,f ) _{j}. \end{aligned}$$

That is,

2.5 $$ \operatorname{div}R\mathring{i}c_{f}=\frac{n-2}{2n} \nabla R_{f}-\frac{1}{2}\nabla\Delta f+\operatorname{div} \mathit{Hess}\,f, $$

where $R\mathring{i}c_{f}=\mathit{Ric}_{f}-\frac{R_{f}}{n}g$.

Then, using

$$\begin{aligned} \int_{M} \langle R\mathring{i}c_{f},hg \rangle \,dv_{f} = & \int _{M} \biggl\langle \mathit{Ric}_{f}- \frac{R_{f}}{n}g,hg \biggr\rangle \,dv_{f} \\ = & \int_{M} ( R_{f}-R_{f} ) h \,dv_{f} \\ = & 0, \end{aligned}$$

we have

2.6 $$\begin{aligned} & \int_{M} ( R_{f}-\overline{R_{f}} ) ^{2}\,dv_{f} \\ &\quad = \int_{M} ( R_{f}-\overline{R_{f}} ) \Delta _{f}u\,dv_{f}=- \int _{M} \langle\nabla R_{f},\nabla u \rangle \,dv_{f} \\ &\quad = \frac{-2n}{n-2} \int_{M} \biggl\langle \operatorname {div}R \mathring{i}c_{f}+\frac{1}{2}\nabla\Delta f- \operatorname{div}\mathit{Hess}\,f,\nabla u \biggr\rangle \,dv_{f} \\ &\quad = \frac{2n}{n-2} \int_{M} \langle R\mathring {i}c_{f}-\mathit{Hess} \,f,\mathit{Hess}_{f}\,u \rangle+\frac{1}{2}\Delta f \Delta_{f}u\,dv_{f} \\ &\quad = \frac{2n}{n-2} \int_{M} \langle R\mathring {i}c_{f}-\mathit{Hess} \,f,\mathit{Hess}_{f}\,u-hg \rangle+\frac{n-2}{2n}\Delta f \Delta_{f}u\,dv_{f} \\ &\quad \leq \frac{2n}{n-2} \Vert R\mathring{i}c_{f}- \mathit{Hess}\,f \Vert _{L^{2}} \Vert \mathit{Hess}_{f}\,u-hg \Vert _{L^{2}}+ \int_{M}\Delta f\Delta _{f}u \,dv_{f}, \end{aligned}$$

where $\Vert \cdot \Vert _{L^{2}}^{2}=\int_{M} \vert \cdot \vert ^{2}\,dv_{f}$ and

2.7 $$ h=\frac{\Delta_{f}u}{n}. $$

Now, we use Bochner’s formula

$$\frac{1}{2}\Delta_{f} \vert \nabla u \vert ^{2}= \vert \mathit{Hess}\,u \vert ^{2}+ \langle\nabla u,\nabla \Delta_{f}u \rangle +\mathit{Ric}_{f} ( \nabla u,\nabla u ) , $$

then

2.8 $$\begin{aligned}& \int_{M} \biggl\vert \mathit{Hess}_{f}\,u- \frac{\Delta_{f}u}{n}g \biggr\vert ^{2}\,dv_{f} \\& \quad = \int_{M} \vert \mathit{Hess}_{f}\,u \vert ^{2}-\frac{ ( \Delta _{f}u ) ^{2}}{n}\,dv_{f} \\& \quad = \int_{M} \vert \mathit{Hess}\,u \vert ^{2}-2 \mathit{Hess}\,u ( \nabla f,\nabla u ) +\frac{ \vert \nabla f \vert ^{2} \vert \nabla u \vert ^{2}+ \langle\nabla f,\nabla u \rangle ^{2}}{2} \\& \qquad {} -\frac{ ( \Delta_{f}u ) ^{2}}{n}\,dv_{f} \\& \quad \leq \int_{M} \biggl( 1-\frac{1}{n} \biggr) ( \Delta _{f}u ) ^{2}-\mathit{Ric}_{f} ( \nabla u,\nabla u ) - \bigl\langle \nabla f,\nabla \vert \nabla u \vert ^{2} \bigr\rangle \\& \qquad {} + \vert \nabla f \vert ^{2} \vert \nabla u \vert ^{2}\,dv_{f} \\& \quad = \int_{M} \biggl( 1-\frac{1}{n} \biggr) ( \Delta_{f}u ) ^{2}-\mathit{Ric}_{f} ( \nabla u, \nabla u ) +\Delta f \vert \nabla u \vert ^{2}\,dv_{f} \\& \quad \leq \int_{M} \biggl( 1-\frac{1}{n} \biggr) ( \Delta _{f}u ) ^{2}+ ( n-1 ) K \vert \nabla u \vert ^{2}\,dv_{f}, \end{aligned}$$

whenever $\mathit{Ric}_{f}\geq ( \Delta f- ( n-1 ) K ) g$.

Since the first positive eigenvalue $\lambda_{1}$ (see [1, 8, 12]) of the weighted Laplacian on *M* is characterized by

$$\lambda_{1}=\inf \biggl\{ \frac{\int_{M} \vert \nabla\varphi \vert ^{2}\,dv_{f}}{\int_{M}\varphi^{2}\,dv_{f}} \Bigm| \varphi \text{ is nontrivial and } \int_{M}\varphi \,dv_{f}=0 \biggr\} , $$

we get

$$\begin{aligned} \int_{M} \vert \nabla u \vert ^{2} \,dv_{f} = & - \int _{M}u\Delta _{f}u\,dv_{f} \\ = & - \int_{M}u ( R_{f}-\overline{R_{f}} ) \,dv_{f} \\ \leq& \Vert u \Vert _{L^{2}} \Vert R_{f}-\overline {R_{f}} \Vert _{L^{2}} \\ \leq& \lambda_{1}^{-1/2}\|\nabla u\|_{L^{2}} \Vert R_{f}-\overline {R_{f}} \Vert _{L^{2}}, \end{aligned}$$

for which it gives the inequalities

2.9 $$ \lambda_{1} \int_{M} \vert \nabla u \vert ^{2} \,dv_{f}\leq \Vert R_{f}-\overline{R_{f}} \Vert _{L^{2}}^{2}\quad \text{and}\quad \lambda_{1}^{2} \int_{M}u^{2}\,dv_{f}\leq \Vert R_{f}-\overline {R_{f}} \Vert _{L^{2}}^{2}. $$

Therefore, (2.8) becomes

2.10 $$ \int_{M} \biggl\vert \mathit{Hess}_{f}\,u- \frac{\Delta_{f}u}{n}g \biggr\vert ^{2}\,dv_{f}\leq A \Vert R_{f}-\overline{R_{f}} \Vert _{L^{2}}^{2}, $$

where

$$A=\frac{n-1}{n}+\frac{1}{\lambda_{1}} ( n-1 ) K. $$

Now, by (2.10), we may rewrite (2.6) as

$$\begin{aligned}& \int_{M} ( R_{f}-\overline{R_{f}} ) ^{2}\,dv_{f} \\& \quad \leq \frac{2n}{n-2} \Vert R\mathring{i}c_{f}- \mathit{Hess}\,f \Vert _{L^{2}} \biggl\Vert \mathit{Hess}_{f}\,u- \frac{\Delta_{f}u}{n}g \biggr\Vert _{L^{2}}+ \int _{M}\Delta f\Delta_{f}u\,dv_{f} \\& \quad \leq \frac{2n\sqrt{A}}{n-2} \Vert R_{f}-\overline {R_{f}} \Vert _{L^{2}} \Vert R\mathring{i}c_{f}- \mathit{Hess}\,f \Vert _{L^{2}}+ \int _{M}\Delta f ( R_{f}-\overline{R_{f}} ) \,dv_{f} \\& \quad \leq \frac{2n\sqrt{A}}{n-2} \Vert R_{f}-\overline {R_{f}} \Vert _{L^{2}} \Vert R\mathring{i}c_{f}- \mathit{Hess}\,f \Vert _{L^{2}}+ \Vert R_{f}- \overline{R_{f}} \Vert _{L^{2}} \Vert \Delta f \Vert _{L^{2}}, \end{aligned}$$

which implies the De Lellis–Topping type inequality

2.11 $$ \Vert R_{f}-\overline{R_{f}} \Vert _{L^{2}}\leq\frac {2n\sqrt{A}}{n-2} \Vert R\mathring{i}c_{f}- \mathit{Hess}\,f \Vert _{L^{2}}+ \Vert \Delta f \Vert _{L^{2}}. $$

If the equality of (2.11) holds, we have the following properties:

(i) $\mathit{Ric}_{f} ( \nabla u,\cdot ) = ( \Delta f- ( n-1 ) K ) g ( \nabla u,\cdot ) $;
(ii) $\mu_{1}(R\mathring{i}c_{f}-\mathit{Hess}\,f)=\mathit{Hess}_{f}\,u-\frac {\Delta_{f}u}{n}g$, where $\mu_{1}$ is a non-zero constant;
(iii) $R_{f}-\overline{R_{f}}=-\lambda_{1}u=\mu_{2}\Delta f$, where $\mu_{2}$ is a non-zero constant;
(iv) $f=\alpha u$, where *α* is constant (since $\int_{M} f\,dv_{f}=0$).

By (iii) and (iv), one has $\Delta_{f}f=\alpha\Delta_{f}u=\alpha\mu _{2}\Delta f$. We rewrite it by

$$( 1-\alpha\mu_{2} ) \Delta f= \vert \nabla f \vert ^{2}, $$

and then it infers that *f* must be zero on *M* since *M* is a closed manifold. Therefore, we complete the proof of Theorem 2.1 by the results of [6] and [11]. □

#### Proof of Theorem 2.2

In the following, we show an almost-Schur lemma under the assumption of *m*-Bakry–Émery Ricci tensor, which is similar to the work of [18]. Consider the nontrivial solution $u:M\rightarrow \mathbb{R}$ of

2.12 $$ \textstyle\begin{cases} \Delta_{f}u=R-\overline{R}, \\ \int_{M}u\,dv_{f}=0, \end{cases} $$

where

$$\overline{R}=\frac{\int_{M}R\, dv_{f}}{V_{f}(M)}. $$

Additionally, the second Bianchi identity $\operatorname{div}\mathit{Ric}=\frac {1}{2}\nabla R$ implies

$$\operatorname{div}R\mathring{i}c=\frac{n-2}{2n}\nabla R, $$

where $( \operatorname{div}\mathit{Ric} ) _{j}=\nabla_{i}R_{ij}$ and $R\mathring{i}c=\mathit{Ric}-\frac{R}{n}g$.

Then we have

2.13 $$\begin{aligned} \int_{M} ( R-\overline{R} ) ^{2}\,dv_{f} = & \int _{M} ( R-\overline{R} ) \Delta_{f}u \,dv_{f}=- \int_{M} \langle \nabla R,\nabla u \rangle \,dv_{f} \\ = & \frac{-2n}{n-2} \int_{M} \langle\operatorname {div}R\mathring {i}c,\nabla u \rangle \,dv_{f} \\ = & \frac{2n}{n-2} \int_{M} \langle R\mathring {i}c,\mathit{Hess}_{f}\,u \rangle \,dv_{f} \\ = & \frac{2n}{n-2} \int_{M} \biggl\langle R\mathring {i}c,\mathit{Hess}_{f}\,u- \frac {\Delta_{f}u}{n}g \biggr\rangle \,dv_{f} \\ \leq& \frac{2n}{n-2} \Vert R\mathring{i}c \Vert _{L^{2}} \biggl\Vert \mathit{Hess}_{f}\,u-\frac{\Delta_{f}u}{n}g \biggr\Vert _{L^{2}}. \end{aligned}$$

Now we use Bochner’s formula

$$\frac{1}{2}\Delta_{f} \vert \nabla u \vert ^{2}= \vert \mathit{Hess}\,u \vert ^{2}+ \langle\nabla u,\nabla \Delta_{f}u \rangle +\mathit{Ric}_{f} ( \nabla u,\nabla u ) , $$

one has

2.14 $$\begin{aligned}& \int_{M} \biggl\vert \mathit{Hess}_{f}\,u- \frac{\Delta_{f}u}{n}g \biggr\vert ^{2}\,dv_{f} \\& \quad = \int_{M} \vert \mathit{Hess}\,u-\nabla f\otimes\nabla u \vert ^{2} -\frac{ ( \Delta_{f}u ) ^{2}}{n}\,dv_{f} \\& \quad \leq \int_{M} \biggl( 1+\frac{m}{2} \biggr) \vert \mathit{Hess}\,u \vert ^{2}+ \biggl( 1+\frac{2}{m} \biggr) \vert \nabla f\otimes\nabla u \vert ^{2}-\frac{ ( \Delta_{f}u ) ^{2}}{n} \,dv_{f} \\& \quad = \int_{M} \biggl( 1-\frac{1}{n}+\frac{m}{2} \biggr) ( \Delta _{f}u ) ^{2}-\frac{m+2}{2} \mathit{Ric}_{f} ( \nabla u,\nabla u ) \\& \qquad {} +\frac{m+2}{2m} \bigl( \vert \nabla f \vert ^{2} \vert \nabla u \vert ^{2}+ \langle\nabla f,\nabla u \rangle ^{2} \bigr) \,dv_{f} \\& \quad \leq \int_{M} \biggl( \frac{n-1}{n}+\frac{m}{2} \biggr) ( \Delta _{f}u ) ^{2}+\frac{ ( m+2 ) ( n-1 ) K}{2} \vert \nabla u \vert ^{2}\,dv_{f}. \end{aligned}$$

Here, we use $\mathit{Ric}_{f}^{m}\geq ( \frac{1}{m} \vert \nabla f \vert ^{2}- ( n-1 ) K ) g$.

Therefore, by inequality (2.9) (but we replace $( R_{f}-\overline{R_{f}} ) $ with $( R-\overline{R} ) $), (2.14) gives

2.15 $$ \int_{M} \biggl\vert \mathit{Hess}_{f}\,u- \frac{\Delta_{f}u}{n}g \biggr\vert ^{2}\,dv_{f}\leq A \Vert R-\overline{R} \Vert _{L^{2}}^{2}; $$

then (2.13) can be rewritten as

2.16 $$ \Vert R-\overline{R} \Vert _{L^{2}}\leq\frac{2n\sqrt{A}}{ n-2} \Vert R\mathring{i}c \Vert _{L^{2}}, $$

where

$$A=\frac{n-1}{n}+\frac{m}{2}+\frac{(m+2)(n-1)K}{2\lambda_{1}}. $$

If the equality of (2.16) holds, then $\mathit{Hess}\,u=\frac{2}{m}\nabla f\otimes\nabla u$ on *M*; which implies

2.17 $$ \Delta_{\frac{2f}{m}}u=\Delta u-\frac{2}{m} \langle\nabla f,\nabla u \rangle=0. $$

That is, *u* is a weighted harmonic function with respect to weighted measure $dv_{\frac{2f}{m}}$ on *M*, it infers $u=0$ on *M*. Thus, Theorem 2.2 follows by the results of [6] and [11]. □

By combining Theorem 2.2 and Theorem 2.1, we note the following property.

#### Corollary 2.1

*Let* $( M^{n},g,dv_{f} ) $, $n>2$, *be a closed smooth metric measure space*. *If*

$$\mathit{Ric}_{f}^{m}\geq \biggl( \frac{1}{m} \vert \nabla f \vert ^{2}- ( n-1 ) K \biggr) Kg $$

*for any positive constant* *m*, *then*

$$\Vert R_{f}-\overline{R_{f}} \Vert _{L^{2}}\leq \frac {2n\sqrt{A}}{n-2} \Vert R\mathring{i}c_{f}-\mathit{Hess}\,f \Vert _{L^{2}}+ \Vert \Delta f \Vert _{L^{2}}, $$

*where* $\Vert \cdot \Vert _{L^{2}}^{2}=\int_{M} ( \cdot ) ^{2}\,dv_{f}$ *and*

$$A=\frac{n-1}{n}+\frac{m}{2}+\frac{ ( m+2 ) ( n-1 ) K}{2\lambda_{1}}, $$

*and* $\lambda_{1}$ *is the first positive eigenvalue of the weighted Laplacian* $\Delta_{f}$. *Moreover*, *the equality holds if and only if* *M* *is Einstein and has constant scalar curvature with respect to metric g*.

### Proof of Theorem 2.3 and partial result

This is similar to the process from (2.12) to (2.15) (in the proof of Theorem 2.2), but we replace (2.14) with the following formula:

$$\begin{aligned}& \int_{M} \biggl\vert \mathit{Hess}_{f}\,u- \frac{\Delta_{f}u}{n}g \biggr\vert ^{2}\,dv_{f} \\& \quad = \int_{M} \vert \mathit{Hess}_{f}\,u \vert ^{2}-\frac{ ( \Delta _{f}u ) ^{2}}{n}\,dv_{f} \\& \quad = \int_{M} \vert \mathit{Hess}\,u \vert ^{2}-2\, \mathit{Hess}\,u ( \nabla f,\nabla u ) +\frac{ \vert \nabla f \vert ^{2} \vert \nabla u \vert ^{2}+ \langle\nabla f,\nabla u \rangle ^{2}}{2}- \frac{ ( \Delta_{f}u ) ^{2}}{n}\,dv_{f} \\& \quad \leq \int_{M} \biggl( 1-\frac{1}{n} \biggr) ( \Delta _{f}u ) ^{2}-\mathit{Ric}_{f} ( \nabla u,\nabla u ) - \bigl\langle \nabla f,\nabla \vert \nabla u \vert ^{2} \bigr\rangle + \vert \nabla f \vert ^{2} \vert \nabla u \vert ^{2}\,dv_{f} \\& \quad = \int_{M} \biggl( 1-\frac{1}{n} \biggr) ( \Delta_{f}u ) ^{2}-\mathit{Ric}_{f} ( \nabla u, \nabla u ) +\Delta f \vert \nabla u \vert ^{2}\,dv_{f} \\& \quad \leq \int_{M} \biggl( 1-\frac{1}{n} \biggr) ( \Delta _{f}u ) ^{2}+ ( n-1 ) K \vert \nabla u \vert ^{2}\,dv_{f}. \end{aligned}$$

Here, we use the curvature assumption $\mathit{Ric}_{f}\geq ( \Delta f- (n-1 ) K ) g$.

Thus, we obtain

2.18 $$ \int_{M} \biggl\vert \mathit{Hess}_{f}\,u- \frac{\Delta_{f}u}{n}g \biggr\vert ^{2}\,dv_{f}\leq A \int_{M} ( R-\overline{R} ) ^{2}\,dv_{f}, $$

and then inequality (2.3)

2.19 $$ \int_{M} ( R-\overline{R} ) ^{2}\,dv_{f} \leq\frac {4n^{2}A}{ ( n-2 ) ^{2}} \int_{M} \vert R\mathring{i}c \vert ^{2} \,dv_{f} $$

holds, where

$$A=\frac{n-1}{n}+\frac{ ( n-1 ) K}{\lambda_{1}}. $$

If the equality of (2.19) holds, we have the properties:

(i) $\mathit{Ric}_{f} ( \nabla u,\cdot ) = ( \Delta f- ( n-1 ) K ) g ( \nabla u,\cdot ) $;
(ii) $\mu R\mathring{i}c=\mathit{Hess}_{f}\,u-\frac{\Delta_{f}u}{n}g$, where *μ* is a non-zero constant;
(iii) $R-\overline{R}=-\lambda_{1}u$;
(iv) $f=\alpha u$, where *α* is constant.

In the following, we prove that if the equality of (2.19) holds under the condition $\alpha\leq\frac{1}{n-1}$, then *M* is Einstein and has constant scalar curvature with respect to metric *g* but remains an open problem whenever $\alpha>\frac{1}{n-1}$.

It is clear that if $\alpha=0$, theorem follows by [6] (or [11] for $K=0$). Therefore, we focus on $\alpha\neq0$.

By (ii), (2.18), and (2.19), we compute *μ* as follows.

2.20 $$\begin{aligned} \mu^{2} \int_{M} \vert R\mathring{i}c \vert ^{2} \,dv_{f} = & \int _{M} \biggl\vert \mathit{Hess}_{f}\,u- \frac{\Delta_{f}u}{n}g \biggr\vert ^{2}\,dv_{f} \\ = & A \Vert R-\overline{R} \Vert _{L^{2}}^{2} \\ = & \frac{4n^{2}A^{2}}{ ( n-2 ) ^{2}} \int_{M} \vert R\mathring{i}c \vert ^{2} \,dv_{f}, \end{aligned}$$

which gives

$$\biggl( \mu^{2}-\frac{4n^{2}A^{2}}{ ( n-2 ) ^{2}} \biggr) \int _{M} \vert R\mathring{i}c \vert ^{2} \,dv_{f}=0. $$

Hence, we have

2.21 $$ \mu=\frac{2nA}{n-2}=\frac{2n}{n-2} \biggl( \frac{n-1}{n}+ \frac { ( n-1 ) K}{\lambda_{1}} \biggr) . $$

By (i) and (iv),

$$R_{ij}u_{,i}+\alpha u_{,ij}u_{,i}-\alpha u_{,j}\Delta u+ ( n-1 ) Ku_{,j}=0 $$

implies

2.22 $$\begin{aligned} \begin{aligned}[b] &R_{ij,j}u_{,i}+\alpha u_{,ijj}u_{,i}-\alpha ( \Delta u ) _{,j}u_{,j}+R_{ij}u_{,ij}+ \alpha u_{,ij}^{2} \\ &\quad {} -\alpha ( \Delta u ) ^{2}+ ( n-1 ) K\Delta u=0. \end{aligned} \end{aligned}$$

Additionally, (ii) gives

2.23 $$\begin{aligned} \mu R_{ij}u_{,ij} = & \biggl\langle \frac{\mu R}{n}g+ \mathit{Hess}_{f}\,u-\frac {\Delta_{f}u}{n}g,\mathit{Hess}\,u \biggr\rangle \\ = & \frac{\mu R}{n}\Delta u+ \vert \mathit{Hess}\,u \vert ^{2}-\alpha \mathit{Hess}\,u ( \nabla u,\nabla u ) \\ &{} -\frac{\Delta u-\alpha \vert \nabla u \vert ^{2}}{n}\Delta u. \end{aligned}$$

Let *u* have minimum at $p\in M$; that is, $u ( p ) =\inf_{M}u$. Then (2.22) and (2.23) become

2.24 $$ \textstyle\begin{cases} R_{ij}u_{,ij} = \alpha ( \Delta u ) ^{2}-\alpha \vert \mathit{Hess}\,u \vert ^{2}- ( n-1 ) K\Delta u, \\ \mu R_{ij}u_{,ij} = \frac{\mu R}{n}\Delta u+ \vert \mathit{Hess}\,u \vert ^{2}-\frac{1}{n} ( \Delta u ) ^{2}, \end{cases} $$

at *p*, for which we have

2.25 $$\begin{aligned} 0 = & \frac{\mu R}{n}\Delta u+ ( 1+\alpha\mu ) \vert \mathit{Hess}\,u \vert ^{2}- \biggl( \frac{1}{n}+\alpha\mu \biggr) (\Delta u ) ^{2}+ ( n-1 ) \mu K\Delta u \\ = & ( 1+\alpha\mu ) \vert \mathit{Hess}\,u \vert ^{2}- \frac{1+\alpha\mu}{n} ( \Delta u ) ^{2} \\ &{} +\frac{\mu}{n} \bigl( R- ( n-1 ) \alpha\Delta u+n ( n-1 ) K \bigr) \Delta u, \end{aligned}$$

at *p*.

Since

$$\begin{aligned} R- ( n-1 ) \alpha\Delta u+n ( n-1 ) K = & \overline {R}+\Delta u- ( n-1 ) \alpha\Delta u+n ( n-1 ) K \\ = & \bigl( 1- ( n-1 ) \alpha \bigr) \Delta u+\overline {R}+n ( n-1 ) K, \end{aligned}$$

(2.25) can be rewritten as

2.26 $$\begin{aligned}& ( 1+\alpha\mu ) \biggl( \vert \mathit{Hess}\,u \vert ^{2}- \frac{1}{n} ( \Delta u ) ^{2} \biggr) +\frac{\mu }{n} \bigl( 1- ( n-1 ) \alpha \bigr) ( \Delta u ) ^{2} \\& \qquad {}+\frac{\mu}{n} \bigl( \overline{R}+n ( n-1 ) K \bigr) \Delta u \\& \quad = 0,\quad \text{at }p. \end{aligned}$$

Because of the curvature assumption

2.27 $$ \mathit{Ric}+\alpha\, \mathit{Hess}\,u\geq \bigl( \alpha\Delta u- ( n-1 ) K \bigr) g, $$

we have

2.28 $$ R\geq\alpha ( n-1 ) \Delta u-n ( n-1 ) K, $$

which gives

2.29 $$\begin{aligned} \overline{R} \geq& \frac{\alpha ( n-1 ) }{V_{f} ( M ) } \int_{M}\Delta u\,dv_{f}-n ( n-1 ) K \\ = & \frac{\alpha^{2} ( n-1 ) }{V_{f} ( M ) } \int _{M} \vert \nabla u \vert ^{2} \,dv_{f}-n ( n-1 ) K \\ > & -n ( n-1 ) K,\quad \text{for all }\alpha\neq0. \end{aligned}$$

Here, we use

$$\Delta u=\Delta_{f}u+\alpha \vert \nabla u \vert ^{2}$$

and integration by parts.

If $\frac{-1}{\mu}\leq\alpha\leq\frac{1}{n-1}$, by (2.29), each term on the left-hand side of (2.26) must be nonnegative at *p*; therefore, $\Delta u ( p ) =0$, which implies $R ( p ) =\sup_{M}R= \overline{R}$, and then *M* is Einstein and has constant scalar curvature with respect to metric *g*.

If $\alpha\leq-\frac{1}{\mu}$, we rewrite (2.26) as

2.30 $$\begin{aligned}& ( 1+\alpha\mu ) \bigl( \vert \mathit{Hess}\,u \vert ^{2}- ( \Delta u ) ^{2} \bigr) +\frac{ ( n-1 ) +\mu }{n} ( \Delta u ) ^{2} \\& \quad {}+\frac{\mu}{n} \bigl( \overline{R}+n ( n-1 ) K \bigr) \Delta u=0 \quad \text{at }p. \end{aligned}$$

We note that at *p*, the $n\times n$ matrix $\mathit{Hess}\,u$ must be semi-positive. Then $\vert \mathit{Hess}\,u \vert ^{2}\leq ( \Delta u ) ^{2}$ at *p*, and the equality holds only if the rank of $\mathit{Hess}\,u ( p ) $ is less than 2. From this inequality, each term on the left-hand side of (2.30) must be nonnegative. Therefore, $\Delta u ( p ) =R ( p ) -\overline{R}=0$, and then *M* is Einstein and has constant scalar curvature with respect to metric *g*.

## Conclusion

This paper contributes two main points. One is that two types of almost-Schur inequalities on smooth metric measure spaces are established under *m*-Bakry–Émery Ricci conditions or ∞-Bakry–Émery Ricci conditions, which imply the results of Cheng [6] and De Lellis–Topping [11] whenever the weighted function *f* is constant. The other is that the equality of our inequality implies geometric qualities of manifold, because the equality holds if and only if the manifold is Einstein and has constant scalar curvature with respect to the background metric (see Theorem 2.1, Theorem 2.2, Corollary 2.1, and a partial result of Theorem 2.3 in Sect. 2.2).

## Methods

In this paper, we show almost-Schur inequalities on smooth metric measure spaces. The key points in the proofs are $\nabla f\otimes\nabla u$ and Bochner’s formula, then due to the Bianchi identity and the first positive eigenvalue of the weighted Laplacian, we establish the almost-Schur inequalities.
